# Public Healthcare for People with Parkinson's Disease in Portugal: Rising to the Challenge

**DOI:** 10.1002/mds.70078

**Published:** 2025-09-29

**Authors:** Sara Varanda, Gisela Carneiro, João Pinho

**Affiliations:** ^1^ Department of Neurology Hospital de Braga Braga Portugal; ^2^ Department of Neurology University Hospital RWTH Aachen Aachen Germany

We read with interest the letter by Carneiro et al,^1^ which is a reflection on private and public healthcare in Portugal, specifically on what concerns people with Parkinson's disease (PD). The for‐profit sector has gained an increasing importance in the healthcare system in Portugal, with an increase of 700% in the number of outpatient consultations between 1999 and 2023 (40% of all outpatient consultations in 2023).^2^ Despite this massive increase of production in the for‐profit sector, the proportion of people ≥16 years with unmet medical needs in the previous 12 months because of excessive waiting time increased from 0.4% (2020) to 1.2% (2024).^2^ This contradicts the premise that private healthcare improves accessibility. In fact, it accentuates accessibility disparities between people who only have the universal public health insurance and people who can afford to pay for an extra private health insurance or directly pay for private healthcare. Throughout the last decades, we experienced a steady increase in the number of patients treated in our movement disorders clinic, despite available specialized offer in the regional private sector.

The authors suggest they were unable to provide the standard of care for patients with PD while practicing in the public system in Portugal. We highlight some limitations that could contribute to this: insufficient number of neurologists (5.8 neurologists per 100,000 inhabitants in Portugal,^2^ in comparison to 9.2 in Europe^3^); migration of neurologists from the public to private sector; existence of a dual‐practice system (often resulting in reduced schedules of medical doctors in the public sector in favor of their private practice); suboptimal number of hospital beds (338 per 100,000 inhabitants in Portugal, in comparison to 511 in Europe^4^); and absence of financial incentives for public hospitals to organize intensive interdisciplinary treatment programs for selected patients.

Despite the increasing demands on the public system, medical departments have the autonomy to organize resources for patient care and to implement patient care projects. We refute the suggestion that the “30‐minute‐twice‐a‐year” paradigm, which the authors refer to, is the default care for people with movement disorders in the public system in Portugal. There are multiple examples in the public sector in which valuable projects for optimal care of people with movement disorders were developed, including: implementation of deep brain stimulation surgery as early as 2002^5^; implementation of enteral infusion therapy programs (which are currently exclusively initiated in public hospitals); weekly slots in the movement disorders outpatient clinics for emergent consultations; Parkinson's day‐clinics; and nursing teams with specific training in movement disorders.

Health care policy makers have the duty to implement policies that avoid the development of a two‐tier health care system. Medical doctors working in the public healthcare setting have the duty to provide optimal evidence‐based care using the available resources and lobby for more resources where they are needed. The public healthcare system in Portugal is still, in our view, the optimal setting to implement the gold standard of care for people with PD and it is imperative that it continues to improve and rise to the challenge.

## Author Roles

(1) Manuscript preparation: A. Writing of the first draft, B. Review and Critique, C Writing the final manuscript; (2) Literature review; (3) Concept.

S.V.: 1B, 1C, 3.

G.C.: 1B, 1C, 3.

J.P.: 1A, 1B, 1C, 2, 3.

## Financial Disclosure for the Previous 12 Months

S.V. received speaking fees from Organon, Lundbeck, and AbbVie. G.C. and J.P. have nothing to report.

## Data Availability

Data sharing is not applicable to this article as no new data were created or analyzed in this study.
